# Unravelling relationships between obesity, diabetes, and factors related to somatosensory functioning in knee osteoarthritis patients

**DOI:** 10.1007/s10067-024-07022-2

**Published:** 2024-06-24

**Authors:** Lotte Meert, Sophie Vervullens, Christiaan H. W. Heusdens, Rob J. E. M. Smeets, Mira Meeus, Michel G. C. A. M. Mertens

**Affiliations:** 1 Research Group MOVANT, Department of Rehabilitation Sciences and Physiotherapy (REVAKI), University of Antwerp, Wilrijk, Belgium; 2 Research School CAPHRI, Department of Rehabilitation Medicine, Maastricht University, Universiteitssingel 40, 6229 ER Maastricht, The Netherlands; 3 Pain in Motion International Research Group (PiM), Brussels, Belgium; 4 Department of Orthopedics and Traumatology, University Hospital of Antwerp, Edegem, Belgium; 5 Faculty of Medicine and Health Sciences, University of Antwerp, Antwerp, Belgium; 6 CIR Clinics in Revalidatie, Eindhoven, The Netherlands

**Keywords:** Adipose tissue, Body mass index, Diabetes mellitus, Glycated hemoglobin, Knee osteoarthritis, Obesity, Pain

## Abstract

**Objective:**

This study explores the association between obesity, diabetes, and somatosensory functioning in patients with knee osteoarthritis (OA), aiming to understand how metabolic conditions are related to pain mechanisms in this patient population. We hypothesized that higher body mass index (BMI), fat mass, and glycated hemoglobin levels (HbA1c) are associated with signs of altered somatosensory functioning.

**Methods:**

A cross-sectional analysis was conducted as part of a larger multicentre prospective cohort study. Data were collected from patients awaiting total knee arthroplasty in Belgium and the Netherlands. Associations between BMI, fat mass, HbA1c, and various pain-related variables were examined employing Pearson and Spearman correlation analyses which were further analyzed with linear regression techniques.

**Results:**

The study included 223 participants. Analysis revealed a significant although weak negative correlation between fat mass and pressure pain thresholds (PPT) at multiple locations, suggesting a link between higher fat mass and increased mechanical hyperalgesia. There were no significant correlations between BMI and pain-related outcomes. HbA1c levels showed very weak positive correlations with pain measures but did not withstand correction for multiple testing.

**Conclusion:**

The findings indicate that fat mass may be closely associated with altered somatosensory functioning in patients with knee OA. However, no significant correlations were found between BMI or HbA1c levels and pain-related outcomes. Future research should focus on longitudinal studies to elucidate the causal relationships and further explore the impact of metabolic factors on pain mechanisms in this patient population.

**Table:** 

**Key Points** *• The findings indicate that fat mass may be closely associated with altered somatosensory functioning in patients with knee OA.*

## Introduction

Osteoarthritis (OA) is a common and disabling condition with growing prevalence and an expected increase in disease burden [1]. A systematic analysis from the Global Burden of Disease Study found that in 2020 approximately 595 million people, or about 7.6% of the global population, were affected by OA. It is projected that by 2050 this number will increase to 642 million [2]. The knee is the most frequently affected site and knee OA is more prevalent in women than in men, particularly symptomatic knee OA. It causes swelling, stiffness, loss of function, and pain, with pain being the dominant symptom and the primary reason for seeking health services [3, 4].

Traditionally, knee OA pain has been categorized as nociceptive pain, originating from tissue damage. However, the correlation between structural abnormalities (measured by radiography or magnetic resonance imaging) and clinical manifestations has been consistently weak [5]. Moreover, approximately 20% of patients report persistent pain following total knee arthroplasty, despite the removal of the primary source of nociception [6]. This discrepancy underscores the complexity of knee OA pain, suggesting the involvement of other pain mechanisms. Emerging research reveals that a distinct subset of patients with knee OA experiences neuroplastic changes within both the peripheral and central nervous systems, leading to altered pain perception, and thus nociplastic pain, in these patients [7, 8].

In knee OA, pro-inflammatory cytokines, such as interleukin-1β (IL-1β), interleukin-6 (IL-6), and tumor necrosis factor-a (TNF-α) play an important role in sensitizing nociceptors, resulting in peripheral sensitization [9]. This can lead to primary hyperalgesia (an increased pain response to a painful stimulus) and allodynia (a pain response to a non-painful stimulus) [10]. Persistent nociceptive input can also lead to neuronal hyperactivity and hyperexcitability in the spinal cord and brain, known as central sensitisation. Central sensitisation, characterized by allodynia and secondary or widespread hyperalgesia, can be present in knee OA patients [10] as well as in patients post-TKA or post-revision TKA [11]. Thus, even without peripheral nociceptive input from the degenerative joint, central sensitization may still occur and be the underlying cause of chronic pain. Elevated levels of inflammation thus may contribute to pain in knee OA. This increased inflammatory state may stem from metabolic conditions, which are not only highly prevalent in knee OA but also contribute to ongoing low-grade inflammation [12].

Metabolic conditions such as obesity and diabetes mellitus (DM) are highly prevalent in patients with knee OA [13, 14]. Both conditions are also linked to the development and progression of knee OA [13, 15]. Excessive metabolites and nutrients, such as lipids and glucose, contribute to an overproduction of adipokines like leptin, resistin, and visfatin. These adipokines increase the risk of OA by stimulating the release of pro-inflammatory mediators, including cytokines, C-reactive protein, and complements [12]. Adipokines can disrupt cartilage homeostasis either by directly inducing degradation of joint structures or by regulating local inflammatory processes [16]. Obesity, DM, and knee OA are associated with low-grade inflammation [16, 17] and evidence shows an association between increased systemic inflammation and higher preoperative patient-reported pain levels in patients with knee OA and altered somatosensory functioning [18]. Consequently, obesity and DM might be involved in pain modulation processes as well.

To the best of our knowledge, no studies have directly investigated the relationship between somatosensory functioning and the presence of obesity or DM in patients with knee OA. Given the common link of low-grade inflammation with obesity, DM, and with altered somatosensory functioning, this study specifically aims to explore how obesity and DM are associated with somatosensory functioning in patients with knee OA. We hypothesize that obesity and DM are linked to alterations in somatosensory functioning in patients with knee OA. Specifically, we propose that higher body mass index (BMI), fat mass, and glycated hemoglobin levels (HbA1c) are associated with signs of altered somatosensory functioning, such as increased pain hyperalgesia, enhanced pain facilitation, diminished pain inhibition, and more pronounced self-reported symptoms of central sensitization.

## Methods

This cross-sectional study was conducted by applying the Strengthening the Reporting of Observational Studies in Epidemiology (STROBE) statement [19]. The protocol is registered at clinicaltrials.gov (NCT05380648).

### Setting

This study is a secondary analysis of a multicentre prospective cohort study aiming to unravel chronic pain following total knee arthroplasty in patients with knee OA [20]. Patients with knee OA awaiting total knee arthroplasty were recruited at the University Hospital of Antwerp and AZ Monica in Belgium, and the Academic Hospital of Maastricht and St. Jans Gasthuis Weert in the Netherlands. After obtaining informed consent, baseline data were collected from March 2018 to July 2022, with assessments conducted four weeks prior to total knee arthroplasty surgery. The ethical committees in both countries approved the study (BE300201319366 and NL6465408618, respectively).

### Participants

Individuals were considered for participation if they were diagnosed with primary knee OA, were awaiting primary total knee arthroplasty, and were 40 years of age or older. Exclusion criteria included having neurological or systemic illnesses that could influence pain perception, such as experiencing neuropathic-like pain symptoms (as identified through patient interviews using the DN-4), neurological diseases like Parkinson's disease or stroke (CVA), and systemic diseases such as rheumatoid arthritis, polymyalgia rheumatica, or cancer. Additionally, participants who were unable to communicate in or comprehend Dutch were excluded. Upon giving informed consent, subjects filled out a range of demographic and symptom-related questionnaires. These questionnaires could be completed electronically via Qualtrics (www.qualtrics.com), or in traditional paper format for patients who were unfamiliar with using a computer. The physical assessments were carried out by two researchers, L.M. or S.V., in the Sensory Functioning Lab (M^2^SENS) at campus Drie Eiken at the University of Antwerp for those in Belgium, and within the orthopedic departments of the Academic Hospital Maastricht and St. Jans Gasthuis Weert for the Dutch patients. The researchers had undergone practical skills training by a researcher with more than 10-year experience in these measurements and employed uniform measurement protocols.

Patients were asked to abstain from first-stage pain medication, coffee, and alcohol 24 h before the physical measurements. For this secondary explorative analysis, all patients with available baseline data were included.

### Outcomes

#### General

Patients had to fill in a *general socio-demographic questionnaire* to acquire information about demographics.

#### Metabolic variables

##### Body mass index (BMI)

Patients’ body weight was measured using an electronic scale and height was self-reported. BMI was calculated as body weight in kilograms divided by squared body height in meters (kg/m^2^). Self-reported weight and height appears to be a valid measure in men and women across different socio-demographic groups [21].

##### Glycated hemoglobin (HbA1c)

To assess glycemic status, HbA1c was measured using the A1CNow^+^ device [22]. This test requires 5 µL of whole blood (1 large drop) obtained with a finger prick procedure and the result was given after 5 min. The A1CNow + test system has shown to have accuracy that matches that of clinical laboratory analyzers [22].

##### Body fat mass

Fat mass was measured using the Bodystat Quadscan 4000 through bioelectrical impedance analysis. Patients lay supine while electrodes positioned on their hand and foot recorded the fat mass percentage. The Bodystat Quadscan 4000 is a reliable method for monitoring body composition. However, its reliability was tested in a different population (people with phenylketonuria), as there is no available data for patients with knee OA [23].

#### Pain-related variables

##### Questionnaires

*Self-reported central sensitization symptoms*

The Central Sensitization Inventory (CSI) was used to assess self-reported central sensitization symptoms. Each question within this inventory is rated on a scale from zero (indicating “never”) to four (indicating “always”) and the CSI is found to be reliable [24].

*Number of pain locations*

Patients were asked to mark all body parts that were painful during the last week to get an idea about pain distribution [25].

*Pain intensity*

The “pain” subscale of the Knee injury and Osteoarthritis Outcome Score (KOOS) was used to assess pain intensity. Scores were converted to percentages and ranged from zero (extreme pain) to 100 (no pain). The KOOS is a valid and reliable measure [26].

##### Measurements

*Mechanical hyperalgesia*

Pressure Pain Thresholds (PPTs) were measured to explore localized as well as widespread mechanical hyperalgesia. A digital algometer (Wagner FDX 25 Force Gauge algometer, Wagner Instruments, Greenwich, USA) was used to assess PPTs. The probe of the algometer (1 cm^2^) was placed perpendicular to the test surface and pressure was increased until the subject reported a feeling of discomfort. PPTs were measured the medial (three centimeters medial from medial edge of the patella), and lateral knee joint-line (three centimeters lateral from lateral edge of the patella), the anterior tibial muscle of the affected leg (five centimeters distal to the tibial tuberosity), the extensor carpi radialis longus (ECRL) muscle  of the non-dominant side (five centimeter distal from lateral epicondyle of the humerus), and the forehead (right above the eyebrow on the affected side). An average of two measurements, separated by a pause of 30 s, was taken for analysis. An algometer used by a trained assessor has been found valid and reliable [27].

*Thermal allodynia*

Thermal rollers (Rolltemp II Somedic Senselab, Sösdala, Sweden) with predetermined temperatures of 25 °C (cold) and 40 °C (heat) were used to detect *thermal allodynia* (THA (heat) and TCA (cold)) at two local sites (medial and lateral joint line affected knee) and a distant site (ECRL muscle of the non-dominant side). The rollers were passed slightly over the skin and after 10 s the patients were asked to report how painful the thermal sensation has been perceived using the numeric rating scale (NRS), where 0 means no pain and 10 indicates unbearable pain. Higher NRS scores indicate higher local (medial or lateral joint line knee) or widespread (ECRL) thermal allodynia. This method is recommended to test abnormal thermal sensations [28].

*Efficacy of ascending pain modulation*

Function of pain facilitating pathways was assessed with* temporal summation* (TS), using a weighted 60 g Von Frey monofilament at a symptomatic site, the medial joint line of the affected knee and a remote site, in the middle at the dorsal side of the wrist of the affected side. The monofilament was applied repeatedly on the skin at a frequency of 1 Hz for 30 s. A metronome was used for application of the required speed. Patients provided a pain rating, using the NRS, after the first and the final stimulus. The difference between these two scores was used for analysis with higher scores indicating enhanced TS. This method of TS evaluation has been found reliable [29].

*Efficacy of pain inhibition*

Function of pain inhibiting pathways was assessed with* conditioned pain modulation (CPM),* using the Q-sense CPM device (Medoc, Israel). First, a temperature corresponding to a pain intensity of 4/10 was identified on the volar side of the wrist of the affected side. This identified temperature, up to a maximum of 46 °C, was used as test stimulus. When a 4/10 on the NRS was not reached, the maximum temperature of 46 °C was used. Following the determination of the test stimulus intensity, the parallel CPM paradigm was initiated. Over a period of 45 s, the individually determined test stimulus was applied to the affected wrist’s volar side. Patients were instructed to verbally rate the intensity of the test stimulus at intervals during its application, specifically at 10 s, 20 s, 30 s, and 40 s. After a 120-s break, a conditioning stimulus was applied on to the non-affected wrist’s volar side for 65 s. The conditioning stimulus had a temperature which was 0.5 °C higher than the test stimulus, with a maximum temperature of 46.5 °C. Twenty seconds after the initiation of the conditioning stimulus, the test stimulus was applied in parallel. Again, the patients had to score their pain on the side of the test stimulus for four times, after 10, 20, 30, and 40 s. If the NRS at 46.5 °C or the mean NRS of the test stimulus were equal to zero, the patient was excluded from the analysis. The absolute CPM effect was calculated as (NRS score during the conditioning stimulus – NRS score during the test stimulus only), and the relative CPM effect as ((absolute score/NRS score during the test stimulus) * 100).

### Statistical analysis

Data were analyzed using Statistical Package for Social Sciences v.29 (SPSS® IBM®, New York, USA).

First, mean and standard deviation were calculated for continuous variables and numbers and percentages for categorical variables. Second, the association between BMI, fat mass, and HbA1c-level and pain-related variables was explored using Pearson correlation analyses (normally distributed data) or Spearman correlation analyses (non-normally distributed data). A Benjamini–Hochberg correction was applied to adjust for multiple testing and the significance level was therefore set to *p* < 0.003 [30]. The arbitrary criteria set by Evans [31] for analyzing correlation strengths was used for interpretation: 0–0.19 very weak, 0.20–0.39 weak, 0.40–0.59 moderate, 0.60–0.79 strong, and 0.80–1.00 very strong relationship. Third, univariable linear regression analyses were conducted for variables that demonstrated significant correlations, using pain-related variables as dependent variables and metabolic variables as independent variables. Age and sex were included as confounding factors [32, 33]. All analyses were complete case analyses.

## Results

### Participants

A total of 223 patients were included. Patient characteristics and an overview of all missing data are summarized in Table 1. Missing data for HbA1c levels and fat mass can be attributed to temporary device deficits. The absence of PPT data at the forehead can be explained by the fact that this variable was added after the study had already started. Missing data for CPM are due either to temporary device deficits or the absence of pain when applying the test stimulus. All other missing data can be attributed to patients not attending measurements or failing to complete questionnaires.

**Table 1:** Patient characteristics

Variable	Mean (SD) or number (%)	*N* Missing (%)
Demographic variables		
Age (y)	65.52 (7.66)	0 (0)
Female sex (%)	111 (49.8)	0 (0)
Metabolic variables		
BMI (kg/cm^2^)	29.99 (5.25)	3 (1.3)
HbA1c (%)	5.60 (0.60)	21 (9.4)
Fat (%)	35.15 (8.88)	91 (40.8)
Pain-related variables		
CSI (0–100)	28.06 (13.14)	12 (5.4)
KOOS subscale pain (0–100)	44.07 (15.31)	12 (5.4)
Number of pain locations (*n*)	3.45 (2.24)	16 (7.2)
PPT tibialis anterior (Newton)	50.89 (24.81)	3 (1.3)
PPT medial knee (Newton)	42.83 (23.71)	3 (1.3)
PPT lateral knee (Newton)	48.06 (26.58)	3 (1.3)
PPT ECRL (Newton)	37.55 (17.24)	3 (1.3)
PPT forehead (Newton)	30.18 (12.73)	38 (17)
TS medial knee (difference in NRS)	1.23 (2.02)	3 (1.3)
TS medial wrist (difference in NRS)	0.98 (1.56)	4 (1.8)
TCA medial knee (0–10)	0.36 (0.96)	4 (1.8)
THA medial knee (0–10)	0.82 (1.46)	4 (1.8)
TCA lateral knee (0–10)	0.27 (0.91)	4 (1.8)
THA lateral knee (0–10)	0.37 (1.09)	4 (1.8)
TCA ECRL (0–10)	0.19 (0.75)	4 (1.8)
THA ECRL (0–10)	0.45 (1.11)	4 (1.8)
CPM relative (%)	9.94 (48.31)	24 (10.8)
CPM absolute	0.31 (1.35)	22 (9.9)

*BMI*, body mass index; *kg/m*^*2*^, kilograms/squared meter; *HbA1c*, glycated hemoglobin; *KOOS*, knee injury and osteoarthritis outcome scale; *PPT*, pressure pain threshold; *ECRL*, extensor carpi radialis longus; *TS*, temporal summation; *NRS*, numeric rating scale; *TCA*, thermal cold allodynia; *THA*, thermal heat allodynia; *CPM*, conditioned pain modulation; *CSI*, central sensitization inventory

### Bivariate analyses

Table 2 gives an overview of the univariate associations. No correlations were found between BMI and pain-related variables. A weak negative correlation was found between fat mass and PPT at all locations. A weak positive correlation was found between fat mass and TS at the skin overlying the medial joint-line of the knee and CSI score.

**Table 2:** Univariate associations

	BMI (kg/m^2^)			Fat mass (%)			HbA1c (%)		
	*N*	Correlation coefficient	*p* value	*N*	Correlation coefficient	*p* value	*N*	Correlation coefficient	*p* value
CSI	210	*r* = 0.11	0.112	124	*r* = 0.20	0.026*	190	*r* = 0.15	0.043
KOOS subscale pain	210	*r* = − .09	0.180	124	*r* = − 0.12	0.182	190	*r* = − 0.03	0.710
Number of pain locations	205	*r* = 0.10	0.148	121	*r* = 0.15	0.106	191	*r* = 0.04	0.585
PPT tibialis anterior	218	*r* = − 0.12	0.089	132	*r* = − 0.37	< 0.001*	202	*r* = − 0.08	0.258
PPT medial knee	218	*r* = − 0.06	0.367	132	*r* = − 0.30	< 0.001*	202	*r* = − 0.14	0.041
PPT lateral knee	218	*r* = − 0.06	0.368	132	*r* = − 0.38	< 0.001*	202	*r* = − 0.05	0.495
PPT ECRL	218	*r* = 0.05	0.434	132	*r* = − 0.31	< 0.001*	202	*r* = − 0.05	0.481
PPT forehead	185	*r* = − 0.10	0.174	107	*r* = − 0.33	< 0.001*	170	*r* = 0.08	0.310
TS medial knee	218	*r* = 0.05	0.462	132	*r* = 0.27	0.002*	202	*r* = 0.05	0.456
TS medial wrist	217	*r* = − 0.05	0.457	131	*r* = 0.09	0.289	201	*r* = 0.03	0.653
TCA medial knee	217	*ρ* = − 0.05	0.462	131	*ρ* = 0.05	0.568	201	*ρ* = 0.00	0.983
THA medial knee	217	*ρ* = − 0.02	0.801	131	*ρ* = 0.03	0.698	201	*ρ* = 0.01	0.876
TCA lateral knee	217	*ρ* = 0.01	0.942	131	*ρ* = 0.08	0.396	201	*ρ* = − 0.03	0.692
THA lateral knee	217	*ρ* = 0.03	0.621	131	*ρ* = 0.07	0.428	201	*ρ* = − 0.03	0.718
TCA ECRL	217	*ρ* = − 0.09	0.190	131	*ρ* = − 0.04	0.625	201	*ρ* = − 0.10	0.163
THA ECRL	217	*ρ* = − 0.09	0.194	131	*ρ* = − 0.11	0.212	201	*ρ* = − 0.04	0.544
CPM relative	197	*r* = 0.07	0.330	117	*r* = 0.15	0.118	182	*r* = 0.01	0.866
CPM absolute	199	*r* = 0.09	0.090	118	*r* = 0.15	0.147	184	*r* = 0.05	0.051

*BMI*, body mass index; *kg/m*^*2*^, kilograms/squared meter; *HbA1c*, glycated hemoglobin; *CSI*, central sensitization inventory; *KOOS*, knee injury and osteoarthritis outcome scale; *PPT*, pressure pain threshold; *ECRL*, extensor carpi radialis longus; *TS*, temporal summation; *NRS*, numeric rating scale; *TCA*, thermal cold allodynia; *THA*, thermal heat allodynia; *CPM*, conditioned pain modulation

^*^ = *p* values remained significant after Benjamini–Hochberg correction for multiple tests, cut-off *p* < 0.003

### Regression analyses

Table 3 provides a summary of all linear regression analyses with fat mass, as independent variable and the seven pain-related variables (CSI, PPT m. tibialis anterior, PPT medial knee, PPT lateral knee, PPT m. ECRL, PPT forehead, TS medial knee) as dependent variables, considering age and sex as confounders. The only significant result observed was the relation of fat mass to PPT on the anterior tibial muscle (*p* = 0.043) and the forehead (*p* = 0.025). Specifically, an increase in fat mass was associated with a decrease in the PPT at the tibialis anterior muscle and the forehead. All other outcomes were not significantly associated with fat mass (*p* > 0.05).

**Table 3:** Univariable regression analyses accounting for age and sex as confounders

Dependent variables	Independent variable				
	Fat mass (%)				
	*B*	95% CI	*p* value	*R*^2^	Adjusted *R*^2^
CSI	0.21	− 0.21, 0.62	0.323		
PPT tibialis anterior	− 0.75	− 1.48, − 0.03	0.043	0.15	0.13
PPT medial knee	− 0.61	− 1.32, 0.10	0.090		
PPT lateral knee	− 0.67	− 1.44, 0.11	0.091		
PPT ECRL	− 0.32	− 0.80, 0.17	0.198		
PPT forehead	− 0.46	− 0.86, − 0.06	0.025	0.11	0.08
TS medial knee	0.02	− 0.04, 0.08	0.548		

*CSI*, central sensitization inventory; *PPT*, pressure pain threshold; *ECRL*, extensor carpi radialis longus; *TS*, temporal summation

## Discussion

Our study identified significant, although weak negative correlations between fat mass and PPTs at the anterior tibial muscle, medial and lateral knee, ECRL muscle, and the forehead. These findings suggest that an increase in fat mass results in lower PPTs at these sites, indicative of heightened sensitivity to mechanical pressure pain. A weak positive correlation was found between fat mass and both TS at the skin overlying the medial knee and CSI scores, suggesting that greater fat mass is associated with increased TS at the medial knee and higher CSI scores (i.e., more self-reported signs of central sensitization). Finally, regression analyses showed that higher fat levels accounted for 13% of the variance in sensitivity to pressure pain at the anterior tibial muscle and 8% at the forehead, as indicated by the R^2^ values. There were no significant correlations observed between BMI and pain-related outcomes. Although HbA1c levels demonstrated very weak positive correlations with pain measures, these correlations did not remain significant after adjustments for multiple testing.

### Interpretation of results

#### Fat mass

To our knowledge, no studies explored the association between fat mass and measures of pain processing in patients with knee OA. However, there is a study exploring the association between self-reported signs of central sensitization, measured with the CSI, and visceral fat in patients with lumbar spinal stenosis [34]. Patients with a high CSI score (CSI ≥ 40) had a significantly higher mean visceral fat area than the low-CSI group (CSI < 40). This is in line with our results that also show an association between high fat mass and higher CSI scores. This can be explained by an increased production of inflammatory cytokines in fat tissue [12, 35]. These inflammatory cytokines make those individuals more susceptible to signs related to central sensitization like hyperalgesia and allodynia [36]. This is partially consistent with our findings, which also identified a link between higher fat mass and mechanical hyperalgesia, but not with thermal allodynia. Interestingly, fat mass was associated with both local and widespread mechanical hyperalgesia. This association can again be explained by the elevated cytokine levels associated with high fat mass and their impact on somatosensory function [18].

#### BMI

Although we hypothesized an association between BMI and pain-related variables, our findings did not reveal any correlations. One previous study explored the relationship between higher BMI, the severity of pain, and the extent of central sensitization in people with hand OA [37]. They found that patients with hand OA with elevated BMI experienced more severe pain not only in their hands but also in their feet and knees/hips. Additionally, these patients noted a greater number of painful body joints throughout the body, more frequent occurrences of widespread pain, and increased central sensitization (lower PPTs at the anterior tibial muscle and enhanced TS). A causal inference-based mediation analysis revealed that the inflammatory biomarkers leptin and high-sensitivity C-reactive protein may mediate the impact of BMI on hand pain and the count of painful body joints, respectively. However, these factors do not appear to mediate central sensitization. In the current study no measures of inflammatory biomarkers were measured, and as such no mediation analysis was performed. Additionally, while BMI generally correlates well with body fat in younger individuals, this correlation tends to be weaker in older adults, possibly due to age-related lean mass loss [38]. Given that our study population is older (i.e., + 40 years old), the relationship between BMI and fat mass may not be as strong, potentially explaining why we observed associations between fat mass and pain-related variables, but not between BMI and these variables. This is particularly relevant as it is primarily the fat mass, or adipose tissue, that produces inflammatory biomarkers, which in turn may influence pain mechanisms [39, 40].

#### HbA1c

Although we expected to find correlations between HbA1c and pain-related variables, after correction for multiple testing no significant correlations were found. A possible explanation could be that the average HbA1c level in our study population was relatively low (5.60%), suggesting that most patients were not experiencing hyperglycaemia. The lack of elevated HbA1c levels may have limited the variability necessary to detect a relationship with altered somatosensory functioning. Although up until now, no direct link between hyperglycaemia and altered pain processing has been identified, a few explanations for a relationship between hyperglycaemia and central pain processing exist [41]. First, hyperglycaemia and related oxidative stress may trigger pain by enhancing glutamatergic transmission that contributes to central sensitisation [42]. Second, hyperglycaemia can alter the function of gamma-aminobutyric acid (GABA), which plays a key role in the inhibition of pain transmission within the spinal cord [43]. Third, chronic hyperglycaemia may trigger pain by activating glial cells implicated in central sensitisation [44].

### Implications for future research and clinical practice

Given the high prevalence of obesity and DM among individuals with knee OA, elucidating the pathways through which these conditions influence pain processing mechanisms could substantially benefit future patients. A promising approach involves measuring combinations of fat mass and HbA1c levels alongside inflammatory markers such as IL-6, TNF-α, and CRP to gain deeper insights. Moreover, conducting longitudinal trials is crucial for establishing causal relationships between metabolic factors and pain processing. These studies could determine whether interventions aimed at reducing metabolic risk factors also alleviate pain symptoms in knee OA and could thus help clinicians to better tailor interventions in clinical practice.

### Strengths

First, the large sample size is one of the strengths of our study. Second, a comprehensive selection of pain-related variables was assessed in order to measure different pain mechanisms including mechanical hyperalgesia, thermal allodynia, and the wind-up phenomenon. Furthermore, we used multiple measurement sites on both the affected and unaffected sides to comprehensively evaluate both local and widespread pain.

### Limitations

Due to the cross-sectional analysis, no information can be given about causality. To elucidate the causal relationships between metabolic factors and central pain processing in patients with knee OA, longitudinal trials are warranted. Additionally, our CPM method, which excluded patients with a NRS score of 0/10 for the test stimulus, may also be a limitation. It is possible that the painful stimulus was insufficient to provoke a CPM response, leading to unexpected findings. Furthermore, due to temporary device deficits during the study enrollment, fat mass had a lot of missing values. Additionally, the absence of established cut-off values prevented our analysis from distinguishing between patients with normal and abnormal somatosensory function, a differentiation that future research should consider.

## Conclusion

Our study provides novel findings demonstrating associations between fat mass and increased pain sensitivity among patients with knee OA. BMI and HbA1c values showed no association with pain-related variables. Future research should focus on longitudinal studies to better understand the causal relationships and explore the impact of metabolic factors on pain mechanisms in this patient population.

## Data Availability

The datasets generated during and analyzed during the current study are available from the corresponding author on reasonable request.
